# System to Detect Racial-Based Bullying through Gamification

**DOI:** 10.3389/fpsyg.2016.01791

**Published:** 2016-11-23

**Authors:** José A. Álvarez-Bermejo, Luis J. Belmonte-Ureña, Africa Martos-Martínez, Ana B. Barragán-Martín, María del Mar Simón-Marquez

**Affiliations:** ^1^Department of Informatics, Universidad de AlmeríaAlmería, Spain; ^2^Department of Economy and Business, Universidad de AlmeríaAlmería, Spain; ^3^Department of Psychology, Universidad de AlmeríaAlmería, Spain

**Keywords:** bullying, cell phone apps, discrimination, gamification, sociogram

## Abstract

Prevention and detection of bullying due to racial stigma was studied in school contexts using a system designed following “gamification” principles and integrating less usual elements, such as social interaction, augmented reality and cell phones in educational scenarios. “Grounded Theory” and “User Centered Design” were employed to explore coexistence inside and outside the classroom in terms of preferences and distrust in several areas of action and social frameworks of activity, and to direct the development of a cell phone app for early detection of school bullying scenarios. One hundred and fifty-one interviews were given at five schools selected for their high multiracial percentage and conflict. The most outstanding results were structural, that is the distribution of the classroom group by type of activity and subject being dealt with. Furthermore, in groups over 12 years of age, the relational structures in the classroom in the digital settings in which they participated with their cell phones did not reoccur, because face-to-face and virtual interaction between students with the supervision and involvement of the teacher combined to detect bullying caused by racial discrimination.

## Introduction

Bullying and cyberbullying, which are formally defined as is the act of harming or harassing, also via IT networks, in a repeated and deliberate manner against an individual who is unable to defend him, are a serious social problem among youth (Olweus, 1993). In addition, the individual and collective repercussions to adolescents known as victimization (Craig et al., 2009) lead to such injury to minors as psychological imbalance (Cook et al., 2010), suicide, health problems (Klomek et al., 2007) and high-risk social behavior (Carmona-Torres et al., 2015). Minors have to be protected from bullying/cyberbullying and the consequences of this type of aggression, as research in Europe, America, and Spain shows the prevalence of victimization of bullying and cyberbullying among students, suggesting that policies for improving the school environment are necessary (Caravaca et al., 2016). This idea is reinforced by the exhaustive work done in 33 countries from 2002 to 2010 with students aged 10–15, which concluded that countries which have made decisions concerning the social problem of bullying and have carried out specific actions in school contexts have reduced both occasional and chronic victimization (Chester et al., 2015). Another recent study included the family along with the school in the bullying/cyberbullying prevention measures (Ortega et al., 2016). Beyond that, even the need to talk about citizenship is suggested, as the relationship between bullying and cyberbullying with homophobia, sexism, racism and other discriminatory situations, which are reinforced and modeled by the adult society, has been demonstrated, and structured action leading to the prevention of this phenomenon must start when schooling begins (Cabra and Marciales, 2015).

It has been attempted to analyze the causes generating bullying (Kim et al., 2006) and its consequences to its student victims (Bender and Lösel, 2011), and an important number of studies have analyzed its prevention (Ttofi and Farrington, 2010). The main purpose of these studies was to design preventive, analytical and intervention instruments for bullying/cyberbullying (Del Rey et al., 2012; Allison and Kirsten, 2015).

Recent research has concentrated more on a type of bullying called social stigma, that is, because of individual characteristics, such as race, weight, gender, social class or sexual orientation (Rosenthal et al., 2015). This type of bullying shares many characteristics with discrimination (Piña and Callejas, 2005), since the abuse originates from the victim pertaining to a deprecated social group. Research on bullying because of racial stigma has been mainly directed at adults, and the problem of bullying at school due to racial stigma has not been well enough studied in academic literature (Rosenthal et al., 2015). In this study, we concentrated on this type of bullying, because Spain is one of the main host countries for immigrants (Pajares, 2009). In this context, many immigrant students in Spain are bullied because of racial discrimination (Ávila, 2013) with disastrous consequences for their development, such as anxiety, or development of aggressive behavior (Smith et al., 2014).

In view of the above, that is the interest in preventing bullying/cyberbullying in schools, including social stigma, and minimizing later victimization in educational environments, the results of antibullying and bullying prevention programs have had only moderate success in changing intimidating behavior and conduct (Ansary et al., 2015). A review of prevention programs in Europe and the United States showed that interventions for bullying were only effective for a week (Pepler et al., 2004), while another concluded that about 20% of interventions did either not cause any change at all or caused changes that were negative (Craig et al., 2010). Recent reviews have suggested that anti-intimidation programs with the best results are the multidisciplinary or “whole-school” approach, which first, accentuates a good social and emotional climate in the school based on solid antibullying philosophy principles (Thornberg, 2011). Second, those maintaining a long-term program extend it to the community and it is externally supervised. And finally, they are constant and firm in applying the strategies when bullying appears (Ansary et al., 2015). Of the main antibullying programs considered effective, the one most used worldwide, is the “Olweus Bullying Prevention Program,” which originated in Norway in 1983, and has been applied in other countries with some variations, such as the “Toronto Project” in Canada in 1991–1993 (Fekkes et al., 2006), and the “South Carolina Project” in 1995 (Limber, 2004) and the “Seattle Project” in 2003–2005 (Bauer et al., 2007) in the United States.

Another widely used program is the “Sheffield Project” which originated in England in 1991–1993 (Smith and Sharp, 1994). These two programs have been jointly implemented in Belgium since 1995–1997 (Smith et al., 2003), and in Spain, “SAVE (*Sevilla Anti Violencia Escolar*)” since 1996–96 (Ortega, 1997) and “ANDAVE (Andalucía Antiviolencia Escolar)” since 1999–2000 (Ortega and Mora-Merchán, 1998). Another two more recent programs, also from an integral perspective, are the “ViSC Program” started in Austria in 2008 (Trip et al., 2015), and also implemented in Turkey in 2012–2014 (Solomontos-Kauntouri et al., 2016) and the KiVA Project from Finlandia in 2006–2009 (Salmivalli and Poskiparta, 2012), which has been used in Estonia (Ginter and Tropp, 2012), Italy, the Netherlands and Whales (Clarkson et al., 2016). One characteristic which makes this project unique is the use of a virtual working environment with games simulating daily life, for constant team monitoring of the project (Kärnä et al., 2013).

But in the last few years, these “whole-school” programs are being enriched by research on preventing bullying which seeks the answers in students, teachers and families (Patton et al., 2015), (Cabra and Marciales, 2015), as bullying prevention programs should be adapted to the cultural context (Olweus, 2010), make use of ICTs related to social sciences and integrate prevention procedures close to adolescent experience (Huitsing and Veenstra, 2012), such as of cell phones (Ruiz and Belmonte, 2014) and gamification strategies (Bishop, 2014; Richter et al., 2015) in school environments (Hanewinkel, 2004; Middaugh and Kahne, 2013).

The term “gamified” refers to the use of videogame design elements in non-game contexts to improve user experience and the ability to hook and motivate (Schoech et al., 2013). A review of the scientific literature on the use of gamification in educational contexts, that is, the use of game design elements, such as including prizes, reward, score charts, credentials, levels, trophies, and so forth (Kapp, 2013), shows widely extended production spanning from studies on their effects on education (Hakulinen and Auvinen, 2014), improved educational quality (Kuo and Chuang, 2016), simulated activities (Rojas et al., 2014), student attention using mobile devices (Su and Cheng, 2015), motivation of different types of students (Hakulinen and Auvinen, 2014), academic scenarios for gamification (Laskowski, 2015), gamification in the search for educational innovation (González and Area, 2013), learning materials for gamification, and evaluation of gamification for motivating students to learn (Ibanez et al., 2014).

In the specific area of bullying, three applications should be highlighted, FearNot (Vannini et al., 2011), a videogame in third person which promotes learning antibullying strategies and which has had good results in detecting bullying by minors (Sapouna et al., 2010), Mii School (Carmona et al., 2011), a 3D videogame for youths in third person which helps detect bullying in schools based on five scenes in which the adolescent has to make choices from different roles (aggressor, victim), and ARBAX-School Bullying (Raminhos et al., 2015), which is a first-person 3D videogame for 16-year-old students, promoting awareness of racial intimidation, but no research at all has been done on this development.

### Context

**FIGURE 1:**
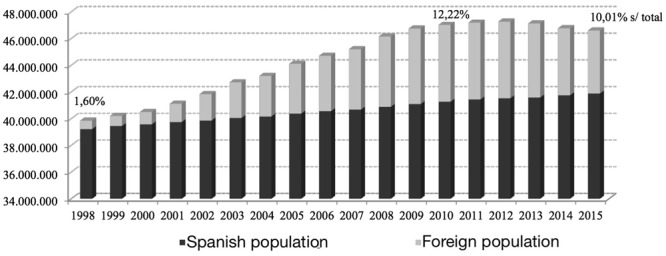
**Evolution of the population: Spaniards and foreigners (1998–2015).** *Source*: INE.

In recent years, as a result of a multitude of complex causes, the immigration role Spain has undergone a radical change since Spain has become one of the main recipients of immigrants, unlike what happened in the 50s and 60s (Pajares, 2009). In the last decade the growth rate of foreigners has been vertiginous, from 1,60% of the population in 1998 to the 12.22% of the population as of January 2012. While from 2012 to 2015 the foreign contingent has been reduced by more than one million people (see **Figure 1**). As a direct consequence of these important migratory movements, many immigrant adolescents under 16 are in the schools.

**FIGURE 2:**
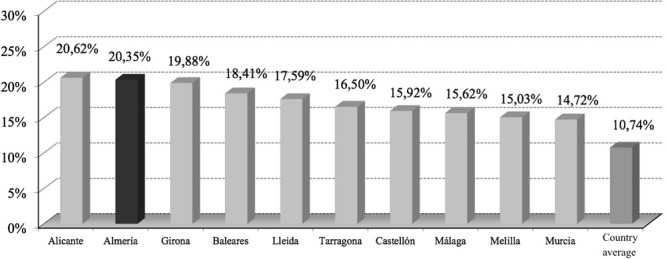
**Provinces with greater weight of foreigners on the total population (2014).** *Source*: INE.

Over the past decade, Spain has experienced a remarkable influx of immigrants, especially sourcing from Africa and from Eastern Europe. In this scenario, Almeria is a priority destination for a bunch of nationalities that, in many cases, are emerging as outsiders with limited financial resources who come to this province in search of a job opportunity, in the sector of the intensive agriculture and sometimes they are discriminated against, relegating them to the last positions of the value chain. More specifically, it is noted that the province of Almeria has shown a tendency of growth of foreign population very pronounced since the mid-nineties, rising from 13,260 in 1998 to 142,810 in 2014 (see **Figure 2**). In this period the foreign population in the province has grown by 977% in simple variation rate, representing an average annual growth rate of 16%. Nationally, the growth of the foreign population between 1998 and 2014, amounted to 689%, in simple variation rate, and 13.8% in cumulative average rate variation. This significance of the contingent of foreigners in the province of Almeria is the highest across the country, behind only of Alicante, which has a percentage of 20.62% of foreigners. In turn, this percentage of significance is almost double the weight that has the group of foreigners in national average (10.74%).

**FIGURE 3:**
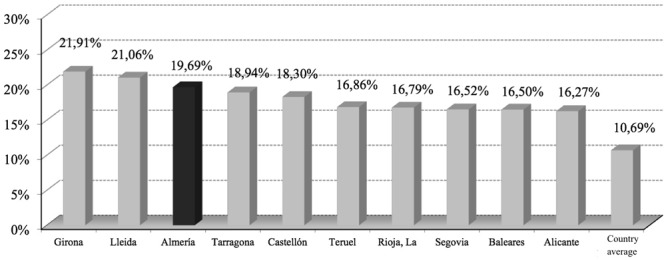
**Provinces with greater weight of under 16 foreigners on the total population of that age group (2014).** *Source*: INE.

If we consider the age group of less than 16 years, it is that the province of Almeria is in third place in the ranking of Spanish provinces with the highest number of adolescent foreigners, on the total population, with 19.69% (**Figure 3**).

**FIGURE 4:**
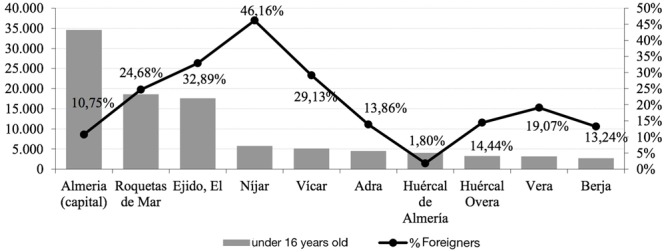
**Municipalities in Almeria with more young people under 16 years (main axis) weight of foreigners on total (secondary axis).** Año 2014. *Source*: INE. **(A)** Database server. Stores the interactions. **(B)** How the interaction works: QR codes identify students. **(C)** All the students are equipped with QR and smartphones. **(D)** Screenshot of the game representing the interaction depicted in **(B)**.

In this regard, at the municipal level, the largest population, under 16, are concentrated around the capital of Almeria, Roquetas de Mar, El Ejido, Níjar and Vícar. These five municipalities account for 64% of young people fewer than 16, of the province (**Figure 4**). Also, in these five territories a large number of young foreigners that, in the case of Nijar, accounts for almost half of the population in this age group. Therefore, under the context depicted, it seems appropriated to analyze the causes of racial harassment stigma among young people under 16 years. That severely affects school life.

## Methodology and Procedure

The review of publications and analysis of antibullying videogames oriented the focus of our research and the methodological design which would best respond to the purpose of developing an application preventing bullying for social stigma. In brief, it showed the need to adapt antibullying action culturally and socially and develop new instruments for social mediation in realistic situations. That is, it should be based on the expressions and experiences of the social actors to find out what preventive/intervention actions, means and measures they prefer. It should also surpass the design of scenarios in which an attempt has been made to monitor the activity of students through the use of traditional videogames (Serrano et al., 2012), which does not provide realistic results since the students are unaware of the real context of their class (i.e., although it is a videogame, it should not detach them completely from their setting). Furthermore, it is understood that it is not appropriate to measure violence using videogames which reinforce it and in which the players do not receive feedback on the consequences of the decisions they make during the game. Therefore, the use of cell phone games based on gamification principles and supported by augmented reality enabling classroom bullying to be detected was the option chosen for this study.

Its methodological design follows the principles of Grounded Theory (Strauss and Corbin, 1998; Charmaz, 2006), as its purpose is to identify basic social processes related to behavior in organizations, groups or social structures such as the school (Glaser, 1992). The grounded theory is very well-established in educational research. Let the work on inclusion in higher education (Givon and Court, 2010), on teaching staff in particular cultural settings (Cherubini et al., 2010), on new teachers during their first year (Smart and Brent, 2010) and in the field of bullying or cyberbullying (Mishna et al., 2009) suffice as examples.

In harmony with the above, User-Centered Design (Norman and Draper, 1986) principles, and specifically for education, Designed Based Research (Luckin et al., 2013), are also followed by incorporating the value of the user in the design of applications and/or tools. However, this subject value does not reside in the traditional view of the user as archetype, client or final user (Maguire, 2001), but in actors, who in this study are bully victims, bullies, witnesses, teachers, families, etc., are not investigated or directed (Sin, 2003; Penuel et al., 2007).

These references help understand the path taken in this study. To explain the methodology more clearly, it is described in two parts. The first part collected information from 151 students at five schools in Almeria province (Spain) with multiracial populations and coexistence problems. As this information is sensitive, it is not available nor has it been published by the schools. Thus their specific selection was based on information acquired from the school direction teams when discussing access with them. Also for reasons of confidentiality, the names of the five schools chosen from among 20 met with are not revealed either. However, **Table 1** shows details on the student population at each school and the characteristics of each group.

**Table 1:** Group characteristics.

Group	School location	Grade	Age	N° of students^1^	% Foreign	Main nationality
A	Almería	1st year high school	12–13	40	8%	Morocco
B	Roquetas de Mar	4th year high school	15–16	27	22%	Rumania
C	El Ejido	5th grade primary	10–11	28	43%	Morocco
D	Níjar	3rd grade primary	8–9	31	39%	Morocco
E	Vícar	2nd grade primary	7–8	25	24%	Morocco

*^1^Number of students in the class at the time of the experiment.*

In addition to the meetings held with the direction teams, informal conversations were held with teachers, students and tutors, some at the school itself, in parents and teachers associations, immigrant associations, parks and plazas. When these were analyzed with the grounded theory (Strauss and Corbin, 1998), categories emerged and by theoretical triangulation (Denzin, 1989), specifically reviewing other questionnaires on school coexistence (Avilés, 2002, un published; Ortega and Del Rey, 2003), an ad hoc questionnaire was designed which was given to 151 students at five selected schools, with items for detecting the existence of discrimination by students, both in the classroom and outside of it (see **Table 2**). All of the items on the questionnaire were measured on a three-point Likert-type response scale where 1 was native student, 2 foreign student and 2 any student. The questionnaires were given a convenience sample of five Spanish students selected from each of the groups.

**Table 2:** Questions asked to detect discrimination in the classroom by students of the mainstream ethnic group.

Multiracial student categories emerged	Information to be collected	Setting
**Knowledge of the other:**
“They don’t respect me” “They don’t know me” “They think I’m stupid” “Sometimes I frighten them” “They think the class is theirs” “They think the playground is theirs “They stare at me”	Identifying the preference/distrust of the nationality of classmates can identify restrictions in developing daily coexistence in the classroom and among students	Coexistence in the classroom
**Knowledge of what separates us:**
“They don’t trust me” “Because we don’t have any money” “They say we smell bad” “Like we’re going to do something to them” “They think they’re better than us” “They think we are all the same”	Identifying the causes of discrimination outside of the classroom can identify restriction of friendship among classmates outside the educational setting	Coexistence outside of the classroom

In the second part of the study, based on the process above, a free cell phone app was developed. This application, designed for smartphones, puts the gamification concept into practice for early detection of bullying among students. Obviously, this application is designed to involve students, but also teachers, since they play a fundamental role in these cases (Dedousis et al., 2014). The application pursues combination of face-to-face and virtual interaction among students, with the supervision and involvement of the teacher, to detect anomalous behavior among the students. It records the interaction among the students to be able to analyze it, classify it and arrive at conclusions. This study did not require ethical approval according to the local legislation. The study was submitted to the ethics committee of the Universidad de Almeria, Spain, who advised that full review and approval was not necessary.

The cell phone app proposal (PREVER, Prevention of Racial Stigma) was designed to evaluate discriminating bias in native youths under 16 against their foreign classmates. The gamified information system we propose is based on an interactive augmented reality game. The student plays, but at the same time he is also part of the game and its real setting (context) along with his classmates. All the students individually and the class as a group are aware that the rest of their classmates are real, as well as the setting, but not the interaction being generated by the game. This way they understand that their actions toward others, although not real, can trigger consequences in the real context. In the game, each participant is identified by a numeric code.

The game architecture is based on each student carrying a cell phone with the app installed. When the students play with the app they see the classroom setting on their cell phones with augmented information. This information comes from identification of the opponents in the application. The whole class has this application and they interact freely with each other. The server collects the data in a csv file (“comma-separated values,” a type of open format document with which it is easy to represent the data in tables) listing all the students so their interaction can be examined in a sociogram.

The interaction model proposed involves evidence of who the interaction takes place between. The participants have information when they interact so that the one who starts it cannot begin without an assumed probable consequence in the real setting. The game also encourages interaction because they are part of a game they are motivated to participate in. Students are identified by a numerical code.

To make it easier to understand, an example of interaction during the game could be that the student is asked to form a group of friends to perform a specific task, organizing a football game. During the interaction, the student is allowed to exclude classmates from the groups with a movement of the phone (the phones have a sensor called an accelerometer which detects how the phone moves and how hard). This way the rejection by one person of another who wants to be on the team is recorded, understanding that not only is it a violent reaction, but segregation. It is attempted to relate this segregating reaction with the difficulty of integrating immigrant students in the schools.

The interaction model not only selects classmates for activities, but can also be played for points two ways, by asking their opponents questions (if their opponent misses, the points are for the attacker) or by pushing (the student makes a pushing motion with the phone, which picks it up and subtracts points from the student attacked. But the one who attacks must answer a question first, and missing takes points away from the attacker).

### System Architecture for the Detection of Racial Harassment Stigma through Gamification

This section describes the architecture of the system designed with the aim of assessing the discriminator bias local youth under 16 years with respect to foreign-born peers described. The gamified information system that we propose is based on an interactive game based on augmented reality. Through this system the student plays, but it is also part of the game and the real environment (context) of the game with his teammates. The game has connections with the real environment, which is an important aspect to consider. Everyone, from the student as the individual to the whole class as the collective, are aware that the other colleagues are real, as the environment. Thus they understand that their actions with others even if they are not real-, can trigger consequences in the real context.

**FIGURE 5:**
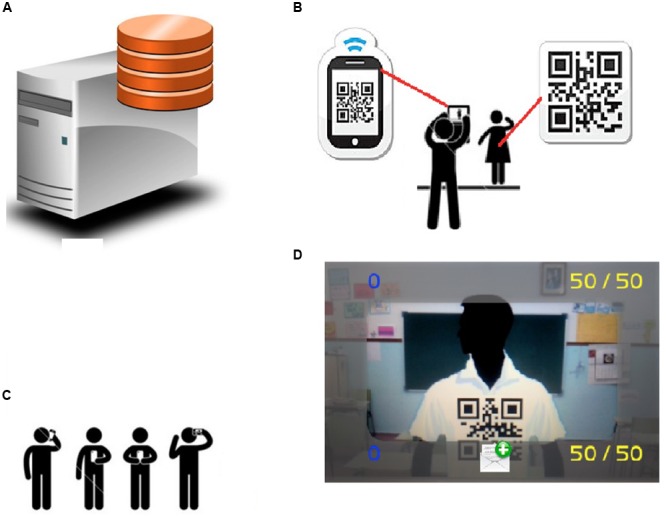
**Proposed architecture**.

The architecture of the game is based on each student carrying two elements (see **Figure 5B**) a Smartphone with the application and a QR code that identifies him/her in the game (each student is put a tag with two QR codes, one in the chest and another in the back). When a child plays with the application, she sees the classroom environment through their mobile device with augmented information (see **Figures 5C,D**). This augmented information comes from the query that the student’s device performs to the terminal server (see **Figure 5A**) using the QR code of the person being pointed by. The server, then, sends interactive information on the student being pointed to the device which it interacts with. The system also recognizes the context in which students (e.g., class, patio) are and whether the interaction is performed through the front QR or through the rear QR code that the student is wearing. The whole class (**Figure 5C**) has these elements and interact freely among them. The server gathers the data in a csv file (comma-separated values; it is a document type in simple open format to represent data in tabular form) relating to all students to be examined by a sociogram (graphical representation of the different relationships between subjects that make up a group). So the interaction analysis can be performed.

The proposed model forces that both parties affected by an interaction are notified of the event. Participants have information notified to them when they are involved in an interaction, so that whoever starts it can not take any action without a supposed and probable consequence in the real environment. Likewise, interactions are catalyzed by the fact that it is a game in which they are motivated to participate.

**FIGURE 6:**
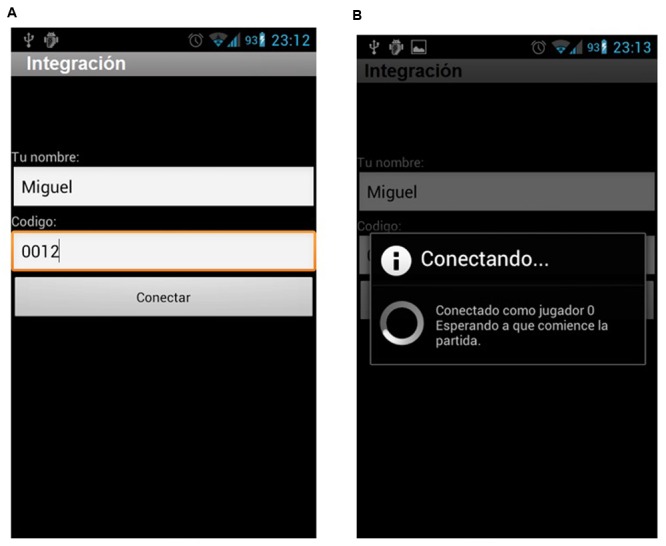
**Smartphone screenshots related to the game. (A)** The student is assigned a numeric Id to avoid identity association. **(B)** The screen shows how, now, student 0012 is connecting. From now, the student will be tagged as 0012 instead of Miguel.

**Figure 6** refers to the starting screen of the game on the student’s smartphone (see **Figure 6A**) and how it connects to the server (see **Figure 6B**) to register the student with his QR codes and data.

**FIGURE 7:**
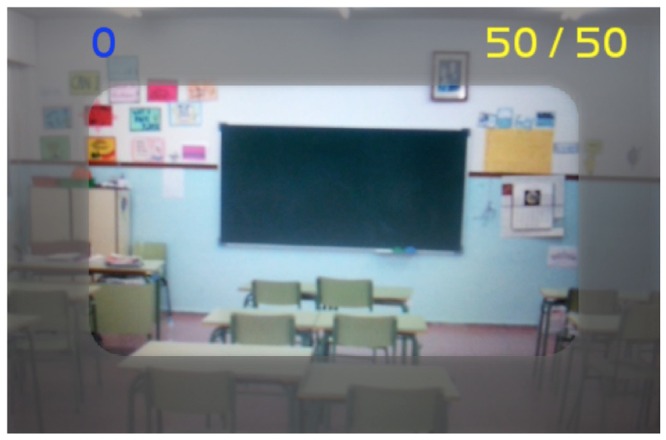
**Mobile screen without selecting a fellow student**.

Following, an example of interaction in which a certain student is required to bringing together his friends in order to perform a task, namely the organization of a football. In interactions it is allowed to exclude fellow students from groups with a movement of the smartphone (smartphones incorporate sensors like accelerometers, which indicates how the phone is moved and how vehemently). Thus the rejection of one person to another who wants to join the team is registered. Understanding that, in this case, the reaction means not only segregation but a violent reaction. It is intended to relate this segregation reaction to the difficulty of integrating immigrant children in schools. **Figure 7** shows the smartphone screen of a student who is not being selected by any partner to perform a specific activity and that is not selecting any other student, he is simply using the game it to observe the classroom. In the top left of the screen you can see the score of the student (depending on the number of people in your group).

**FIGURE 8:**
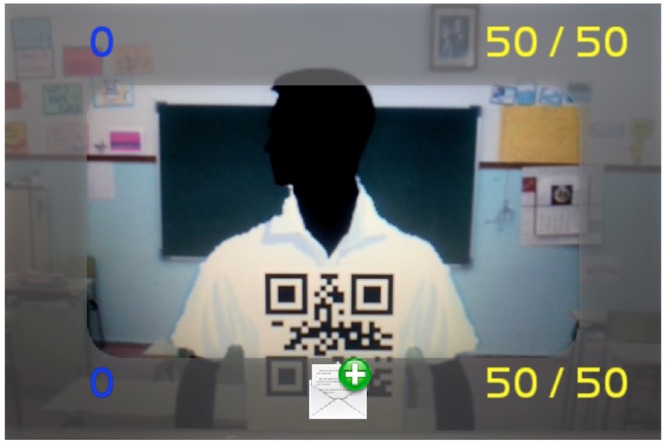
**Mobile screen selecting a fellow student**.

**Figure 8** shows an example of interaction where you can add components to your group once launched a group activity.

**FIGURE 9:**
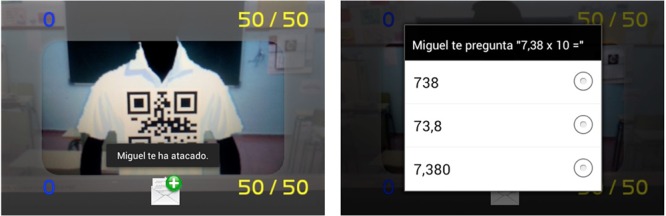
**Validation of an expulsion by the student #0012**.

**FIGURE 10:**
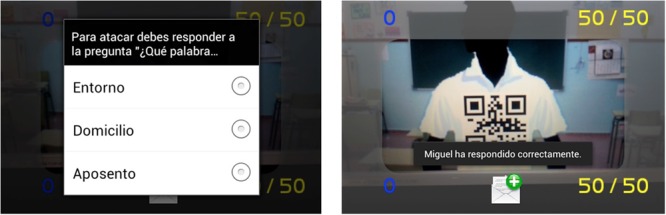
**Cancelation of an expulsion by the student #0012**.

The interaction model is not only limited to directly selecting partners for activities, and in addition it can be controlled in the sense that for every action you want to perform, a question must be correctly answered. In this case, if the question posed at the partner is not resolved, the opponent wins. On the contrary, if resolved who starts the interaction wins (and therefore the action is performed). **Figures 9** and **10** show this feature.

**FIGURE 11:**
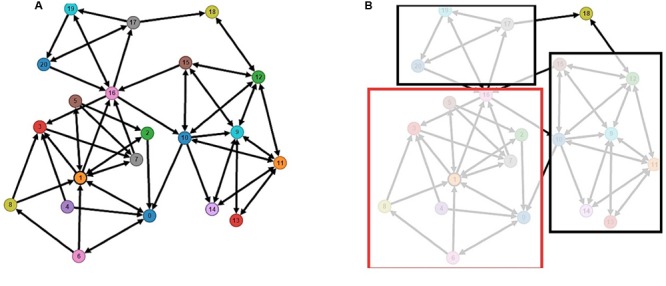
**Sociogram of the interaction. (A)** The complete sociogram of the interaction. **(B)** The sociogram after the racial analysis. We can see how students group by nationality.

As noted above, the application server (see **Figure 5A**) records all interactions between students, so the teacher can access this server to download this information and to control what happens (i.e., what kind of interaction it is generated) among students in the class through a sociogram or x-ray interactions that occur in the group. See **Figure 11**, this is an example of the visual data that the application generates.

## Results

Concerning coexistence in the classroom, it should be mentioned that in four of the five classrooms analyzed, none of the foreign students sat at desks in the front row, that is, they were rather near the back of the class, except in the group of second-graders (Vicar), who were from seven to 8 years old. This agrees with studies done on discrimination in classrooms (i.e., they tend to “hide” in the last rows of the classroom) except in second-grade. Even though descriptive analyses of the data collected from the five schools shows that most of the students prefer to work or share a table with a native classmate (72% of the sample), distrust was not associated to any greater extent with foreign students (60% of the sample said they distrusted any classmate of whatever nationality). With respect to coexistence outside of the classroom, it should be mentioned that discrimination for racial stigma does not seem to be present in sports as clearly as in other cases mentioned above (only 20% of the sample said they would not invite an immigrant classmate). When organizing other non-sport activities (a birthday party), the percentage of rejection of foreign students rises slightly to 24% (64% of the sample said they might not invite “any classmate”). Finally, outside of the classroom in matters leading to closer involvement in their relations (go on vacation with a classmate), the results show that in this case in particular the majority of students prefer to go with foreign students (56% of the sample).

The PREVER app records all the interactions among students as described above, so the teacher can access the server and download this information to monitor what is happening (i.e., what type of interaction is being generated) among the students in the class on a sociogram of interactions going on in the group. **Figure 10** below shows an example of interaction in a class of students in the last year of primary school (to help understand the graphic, unimportant interactions have been omitted and only the more significant are kept in). It may be seen that three differentiated groups are joined by hubs common to them: (1) the group of Spaniards (numbered 0–8) has little interaction with the rest of the students who belong to other groups, (2) groups 9–15 are made up of Moroccans who have also established strong hubs basically among themselves (perhaps due to family interaction and the language, but they refuse to enlarge their groups), and (3) the Romanian students (groups 17, 18, 19, and 20) and one Eastern European student (Russian). This sociogram shows how the students in a class relate to each other. Some strong cores of students and others not as strong are observed. The clusters detected are by origin. It is assumed that as they have the same origin and share the same language, relations among their parents are quite probably closer and this generates stronger bonds.

## Discussion and Conclusion

It is a fact that the discriminatory and excluding behavior in society should be detected early for better coexistence among the various nationalities that make up the population of a territory. However, acceptance of interculturality in the classroom is a problem that arises and affects immigrant students who are discriminated because of their origin and their appearance, which stigmatizes their future personal and professional development (Baysu et al., 2013). Gradual general awareness of the importance of human rights and repercussion in communications media of aggression in schools demand that the parties involved in education intervene in both prevention and treatment of bullying when it occurs. In this sense, the subject of bullying has awakened growing interest in developing prevention and intervention programs for its reduction (Breakstone et al., 2009) which have had ambiguous results. This study has therefore attempted to advance in this matter by offering a gamified cell phone app which assists teachers in finding out the extent of interaction among students in a class and for early detection of possible bullying or discrimination based on the data stored in the proposed system’s server.

The data analyzed demonstrate that the main problems of discrimination affecting the foreign population under 16 years of age in their compulsory education and which could potentially generate bullying are related more to their relations within the classroom itself. In spite of this, the app guides those students who install it on their devices through a series of stages dominated by situations in which they have to choose classmates which will lead them to select those with whom they prefer to interact and relate in each case. Thus it provides a useful dynamic tool for finding out the interactions taking place among students and for detecting cases of discrimination and even extreme cases of bullying.

The main implication of the study is possible decrease in cases of abuse derived from their early detection and consequent intervention/treatment by the teacher (and other parties involved), who can periodically consult data on student interaction stored on the system’s server. It is an application mainly for implementation in classrooms to be used in student leisure periods (e.g., recess) and even outside of the classroom because of its gamified design. However, one of the main lines of future research is integration of the app in the learning setting itself, proposing this new context for interaction among students which could provide more clues to their relationships in the classroom and outside it. Finally, the first thing that should be pointed out concerning the main limitations for using this type of technologies in the classroom is the need for the students to have access to a cell phone in which to install the app developed and interact with their classmates, as well as the different degrees of involvement developed by the students in their use of the applications. Other limitations related to the performance of the teacher’s work should also be mentioned, since the introduction of this type of technology in the classroom means that more time would have to be devoted to periodic control of the interactions among students with the data stored on the server.

## Author Contributions

JA-B: He has developed and designed the software application for detecting the discrimination in the educational context. LB-U: He has been responsible for design the questionnaire that was passed to students in schools, as well as the study of the number of immigrants in Spain and Almeria. AM-M: She has participated in the field study, as pollster. Also, she has participated in the methodological support. AB-M: She has participated in the field study, as pollster. Also, she has participated in the methodological support. MdMS-M: She has participated in the field study, as pollster. Also, she has participated in the methodological support.

## Conflict of Interest Statement

The authors declare that the research was conducted in the absence of any commercial or financial relationships that could be construed as a potential conflict of interest.
